# Unleashing the power of retatrutide: A possible triumph over obesity and overweight: A correspondence

**DOI:** 10.1002/hsr2.1864

**Published:** 2024-02-05

**Authors:** Muhammad Naeem, Laiba Imran, Umm E Salma Shabbar Banatwala

**Affiliations:** ^1^ Department of Medicine Dow University of Health Sciences Karachi Pakistan

**Keywords:** LY3437943, incretin mimetic agents, obesity, overweight, retatrutide, weight‐loss

## Abstract

**Background and Aims:**

Overweight and obesity have become global health challenges with increasing prevalence. Several drugs have received Food and Drug Administration approval for nonsyndromic obesity treatment, but most have limitations, including gastrointestinal side effects and limited weight loss efficacy.

**Body:**

Retatrutide, a novel incretin mimetic agent, has shown promise in clinical trials for significant weight reduction. It has demonstrated dosage‐dependent pharmacokinetics with favorable safety profiles. The primary focus of this paper is to explore retatrutide and critically assess its clinical trials to justify its use and feasibility while highlighting its shortcomings. This paper also delves into the subject of obesity and its health manifestations.

**Conclusion:**

It is expected that the use of retatrutide, a triple agonist, will result in significant weight loss among individuals who are obese or overweight.

Overweight and obesity are characterized by the atypical or disproportionate buildup of fat in the body leading to notable health hazards. As per the World Health Organization (WHO), individuals cross the threshold into overweight territory when their body mass index (BMI) is more than 25 kg/m². Furthermore, a BMI surpassing 30 is indicative of obesity.^1^ Recent statistics from 2022 reveal that globally, the number of obese individuals exceeds one billion and these figures are continuously rising.^2^ The World Obesity Federation's 2023 Atlas presents alarming projections indicating that within the next 12 years, over four billion individuals, constituting 51% of the global population, will be either obese or overweight.^3^ Initially, obesity was thought to result from an imbalance between caloric intake and energy expenditure. However, current understanding recognizes that the development of obesity is influenced by complex interactions of biological and psychosocial factors. For example, various research groups have identified many genetic loci associated with weight and body dimensions. Additionally, rare genetic disorders like Prader–Willi syndrome, characterized by hyperphagia, frequently contribute to obesity.^4^ Not only do overweight and obesity lead to increased fat mass and body surface area, but it also creates an environment conducive to the development of severe diseases. These include heart diseases, diabetes mellitus, stroke, and pregnancy‐related complications.^1^, ^5^

To combat obesity, the most effective approach is weight loss.^6^ Even a modest weight reduction of 5%–10% can yield cardiovascular health benefits and lower blood sugar while >10% weight loss is associated with even greater improvement.^7^, ^8^ Weight management strategies encompass various interventions, starting with dietary choices, physical exercise, and progressing toward cognitive‐behavioral techniques, pharmacological options, and, in some cases, surgical interventions.^9^

There are numerous drugs used as weight‐reducing agents. The regimens recognized by the United States Food and Drug Administration (FDA) for prolonged use for nonsyndromic obesity are orlistat, phentermine‐topiramate, naltrexone‐bupropion, liraglutide, and semaglutide. Two FDA‐accepted medications; setmelanotide, and metreleptin, target individuals diagnosed with monogenic syndromic obesity.^7^, ^10^ Most of them have adverse effects of a gastrointestinal nature such as abdominal pain, nausea, vomiting, diarrhea, or constipation and do not achieve more than 15% weight decrease except with glucagon‐like peptide 1 receptor (GLP‐1R) agonists like semaglutide.^10^, ^11^ While these medications are generally considered safe, some medications carry specific warnings, such as contraindications in individuals with heart and kidney disease, uncontrolled hypertension, glaucoma, hyperthyroidism, or medullary thyroid carcinoma.^10^ A novel medication under investigation is retatrutide, which is an incretin mimetic agent. Incretins decrease appetite and enhance feelings of fullness while slowing gastric emptying (GE). The fewer side effects and high accuracy of incretin mimetic drugs such as liraglutide and semaglutide are opening the door for the development of such drugs.^10^, ^12^

Retatrutide (LY3437943), a single peptide conjugated to a lipid diacid molecule, exerts a powerful agonist effect on the human glucagon‐receptor (GCGR), GIPR, and GLP‐1R. In comparison with the human glucagon and glucagon‐like peptide 1 (GLP‐1), retatrutide exhibits reduced potency (by a factor of 0.3 and 0.4, respectively) on the GCGR and GLP‐1R, while displaying enhanced potency at the human GIPR (by a factor of 8.9) when compared to the glucose‐dependent insulinotropic polypeptide (GIP). In vitro, Retatrutide demonstrates similar efficacy to natural glucagon in evoking glucose production within hepatocytes. While in adipocytes, it surpasses native GIP in inducing lipolysis.^13^ The data from the trials mentioned in this manuscript indicates that Retatrutide is effective in reducing weight in individuals with nonsyndromic obesity.^12^, ^14^, ^15^ Retatrutide demonstrates dosage‐dependent pharmacokinetics, featuring a favorable half‐life of nearly 6 days, thereby enabling convenient weekly administration.^14^ Additionally, GLP‐1 and GCG are known to significantly delay GE in humans, while GIP may have no impact on GE. Delayed GE reduces food consumption and therefore leads to weight reduction.^16^

The weight‐reducing effects of retatrutide have been supported by different clinical trials, presenting compelling evidence. In a randomized, double‐blind, and placebo‐controlled phase 2 study conducted in the United States,^12^ involving obese or overweight, patients were administered subcutaneous retatrutide or placebo weekly for nearly 11 months. The study patients were allocated in a randomized ratio of 2:1:1:1:1:2:2 to receive retatrutide at various doses (1, 4 mg [commencing with 2 mg], 4 mg [commencing with 4 mg], 8 mg [commencing with 2 mg], 8 mg [commencing with 4 mg], or 12 mg [commencing with 2 mg]). A total of 338 adults were enrolled from May 2021 to November 2022, of whom 51.8% were men. In evaluating the primary endpoint, the least‐squares mean weight change at week 24 demonstrated a reduction of −7.2% in the 1‐mg cohort, −11.8% in the 4‐mg group (initiated with 2 mg), −13.9% in the 4‐mg group (commenced with 4 mg), −16.7% in the 8‐mg group (started with 2 mg), −17.9% in the 8‐mg group (initiated with 4 mg), and−17.5% in the 12‐mg group (commenced with 2 mg), in contrast to a mere −1.6% in the placebo group at the same time point. The least‐squares mean percentage shift in weight after 48 weeks (a secondary endpoint) exhibited a decrement of −8.7% in the 1‐mg cohort, −16.3% in the 4‐mg group (commenced with 2 mg), −17.8% in the 4‐mg group (initiated with 4 mg), −21.7% in the 8‐mg group (initiated with 2 mg), −23.9% in the 8‐mg group (commenced with 4 mg), and −24.2% in the 12‐mg group (initiated with 2 mg), in contrast to a meager −2.1% in the placebo group at the designated 48‐week interval. After 48 weeks, an impressive percentage of participants (64%–100%) allocated to treatment with retatrutide successfully attained weight loss of 5% or greater, in comparison to only 27% of participants in the control group. More than a quarter (26%) of participants got rid of 30% or more of their baseline weight, in the 12‐mg retatrutide group. Weight reduction was greater in more obese men >35 BMI than those who were under 35 BMI and also among females than males. The results also showed significant reductions in waist circumference for participants receiving retatrutide of up to −19.6 cm, compared to −2.6 cm in the control group. Analysis at weeks 24 and 48 showed that retatrutide participants have gained significant improvements in cardiometabolic measures such as levels of hemoglobin A1c (HbA1c), fasting blood glucose, insulin, systolic and diastolic blood pressure, and lipids (except high‐density lipoprotein [HDL]). At week 48, a significant majority (72%) of participants in the intervention groups achieved normoglycemia (HbA1c < 39 mmol/mol or <5.7%), having initially presented with prediabetes during recruitment. In contrast, only 22% of participants in the placebo group attained normoglycemia. Notably, only 1% of subjects exposed to retatrutide experienced a transient elevation in alanine aminotransferase (ALT) levels, surpassing three times the upper limit of the normal range. Nevertheless, as they reached the 48th week, the mean levels of ALT and aspartate aminotransferase (AST) remained in a state of equipoise or even manifested a discernible decrement. In this trial^12^ at 24 weeks, the increments in heart rate were dose‐dependent, reaching a peak and declining thereafter. Supraventricular arrhythmias and cardiac conduction disorders were noticed in a few participants which were not serious. Among the participants, a total of 15 serious adverse events were reported, affecting 13 individuals. Notably, the frequencies of these events were similar in both the retatrutide and placebo groups, with a prevalence of 4% in each group.^12^

In a phase 1 randomized clinical trial^14^ conducted between December 18, 2019, and December 28, 2020, in the United States, with 72 individuals diagnosed with diabetes mellitus type 2, the administration of weekly subcutaneous injections of 12‐mg retatrutide led to a remarkable average weight loss of 8.96 kg (nearly 10%). In this multicenter trial, retatrutide exhibited a favorable safety profile and its pharmacokinetics indicate that it is well‐suited for weekly administration in type 2 diabetics. Retatrutide also demonstrated reductions in glycated hemoglobin (HbA1c) of −1.2% [90% confidence interval: −2.05 to −0.45] for 3/6/9/12 mg) compared to placebo and dulaglutide after 12 weeks of treatment. Additionally, elevated insulin levels were noted alongside a decrease in systolic blood pressure. Furthermore, there were reductions in low density lipoprotein  cholesterol, triglycerides, and very low density lipoprotein cholesterol compared to the placebo, with no significant reduction in HDL cholesterol observed with the placebo.^14^ In another randomized, double‐blind phase 2 trial,^15^ conducted between May 13, 2021, to October 27, 2022, in the United States a total of 281 people were recruited and randomly allocated with categorization based on both baseline BMI and HbA1c levels to receive once‐a‐week shots of a placebo, varying retatrutide dosages, or 1.5 mg dulaglutide using a syringe or single‐dose pen. The decrease in body weight was most pronounced in the 4 mg and greater dosage groups of retatrutide compared with placebo (*p* = 0.0017 for the 4 mg escalation group and *p* < 0.0001 for others) and 1.5 mg dulaglutide (all *p* < 0.0001) at 36 weeks. Additionally, a −2.02% (0.11; −22.07 mmol/mol [1.21]) difference in HbA1c levels was reported in the 12‐mg retatrutide group contrasting to a ‐ 0.01% (0.21; −0.12 mmol/mol [2.27]) or difference in the control group. At the 36‐week mark, the utilization of retatrutide engendered a notable enhancement in insulin sensitivity. On the whole, the mean values of ALT and AST exhibited a discernible decline from their initial baseline levels during the course of retatrutide therapy.^15^ In one trial,^12^ there is a weight reduction of 30% or more, whereas in another trial,^14^ the reduction is nearly 10%. Ongoing phase 3 trials, known as Triumph trials,^17^ are evaluating the long‐term safety profile and effectiveness of retatrutide for lowering weight, osteoarthritis, and obstructive sleep apnea in obese, overweight, or type 2 diabetic individuals. Other GIP‐GCG‐GLP‐1 receptor agonists, such as HM15211 and SAR441255, are also undergoing trials for obesity and similar conditions.^18^, ^19^

The findings from these studies unveiled that the predominant treatment‐emergent adverse events linked to retatrutide are mild or moderate gastrointestinal issues, like nausea, vomiting, diarrhea, and constipation. Importantly, these events demonstrated a dependency on the dosage administered.^12^, ^14^, ^15^

These studies^12^, ^14^, ^15^ were conducted on a relatively small sample size and predominantly on a geographically homogeneous population (United States), which restricts the generalizability of the results to a bigger population with diverse ethnicities. Moreover, the duration of these trials was insufficient to evaluate the longer‐term effects of retatrutide on weight loss. Furthermore, the benefits along with the potential risks of using this drug for other health conditions have not been established. Long‐term safety profiles and efficacy data are also lacking, emphasizing the need for further studies and trials in the future. It is crucial to conduct phase 3 trials with participants from diverse ethnic backgrounds to assess long‐term efficacy, safety profiles, and adverse events. We encourage specialists to conduct essential research to ensure our expectations are well‐informed.

In conclusion, it is expected that the use of retatrutide, a triple agonist, will result in significant weight loss among individuals who are obese or overweight. This medication has the potential to revolutionize pharmacotherapy for obesity and overweight in the coming years. Nonetheless, additional research and clinical trials are imperative to comprehensively grasp the treatment's long‐term impacts, safety, and effectiveness.

## AUTHOR CONTRIBUTIONS

**Muhammad Naeem**: Writing—original draft. **Laiba Imran**: Conceptualization; writing—review and editing. **Umm E Salma Shabbar Banatwala**: Writing—original draft; writing—review and editing.

## CONFLICT OF INTEREST STATEMENT

The authors declare no conflict of interest.

## TRANSPARENCY STATEMENT

The lead author Umm E Salma Shabbar Banatwala affirms that this manuscript is an honest, accurate, and transparent account of the study being reported; that no important aspects of the study have been omitted; and that any discrepancies from the study as planned (and, if relevant, registered) have been explained.

## Data Availability

The article does not use any original data which is to be reported.
